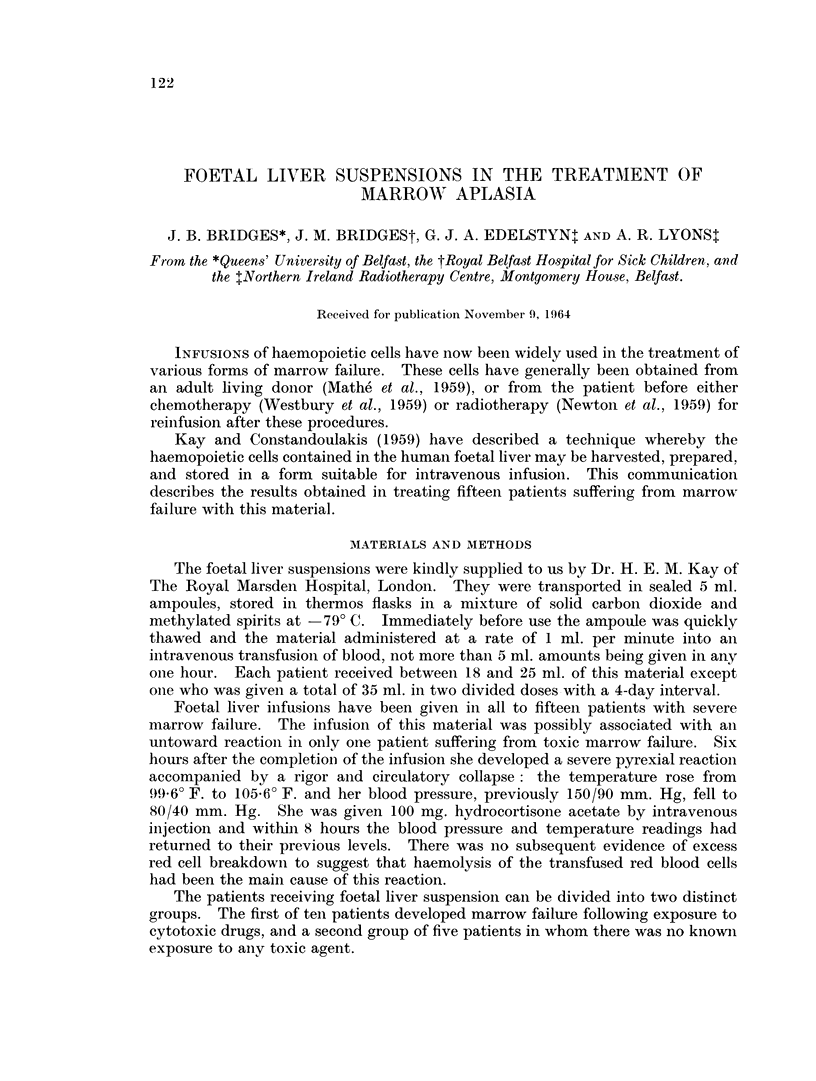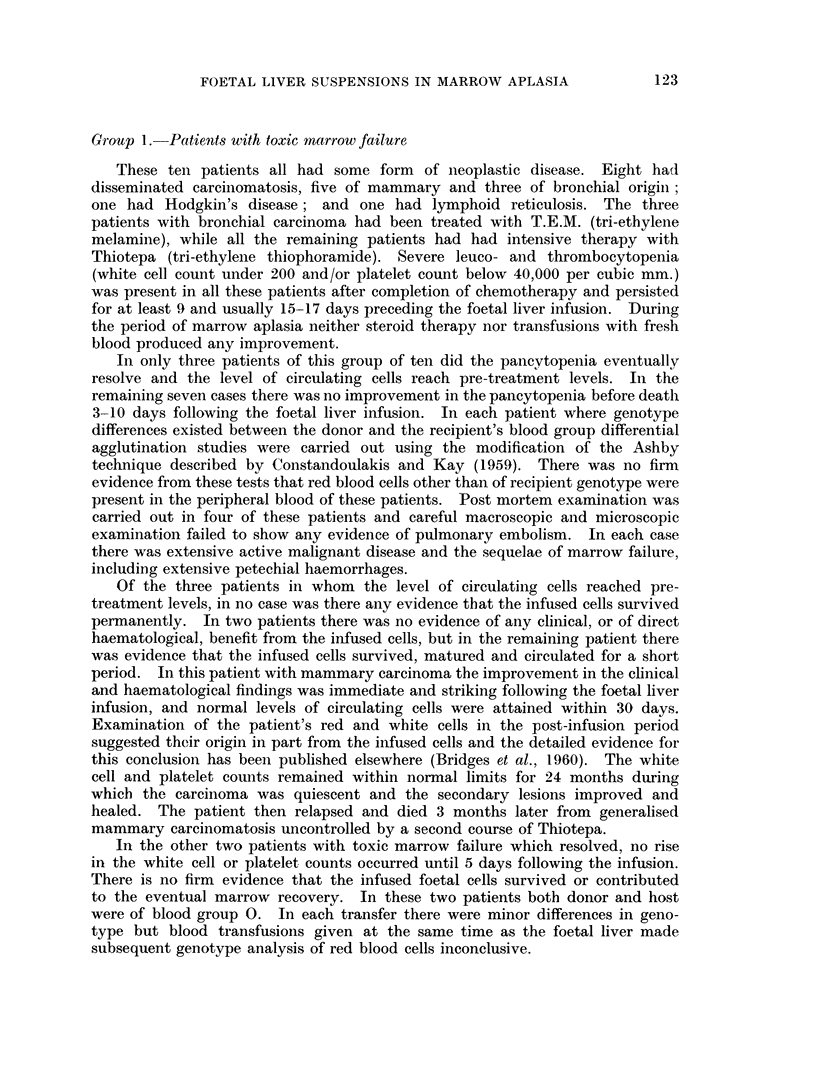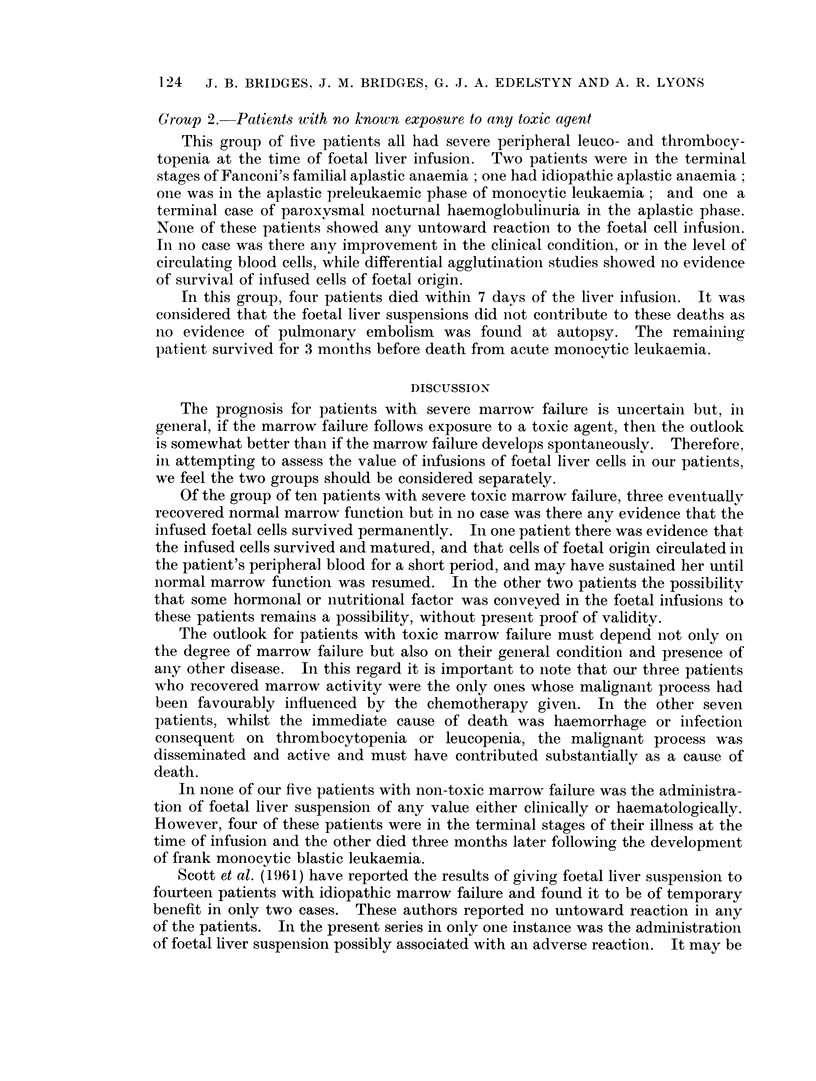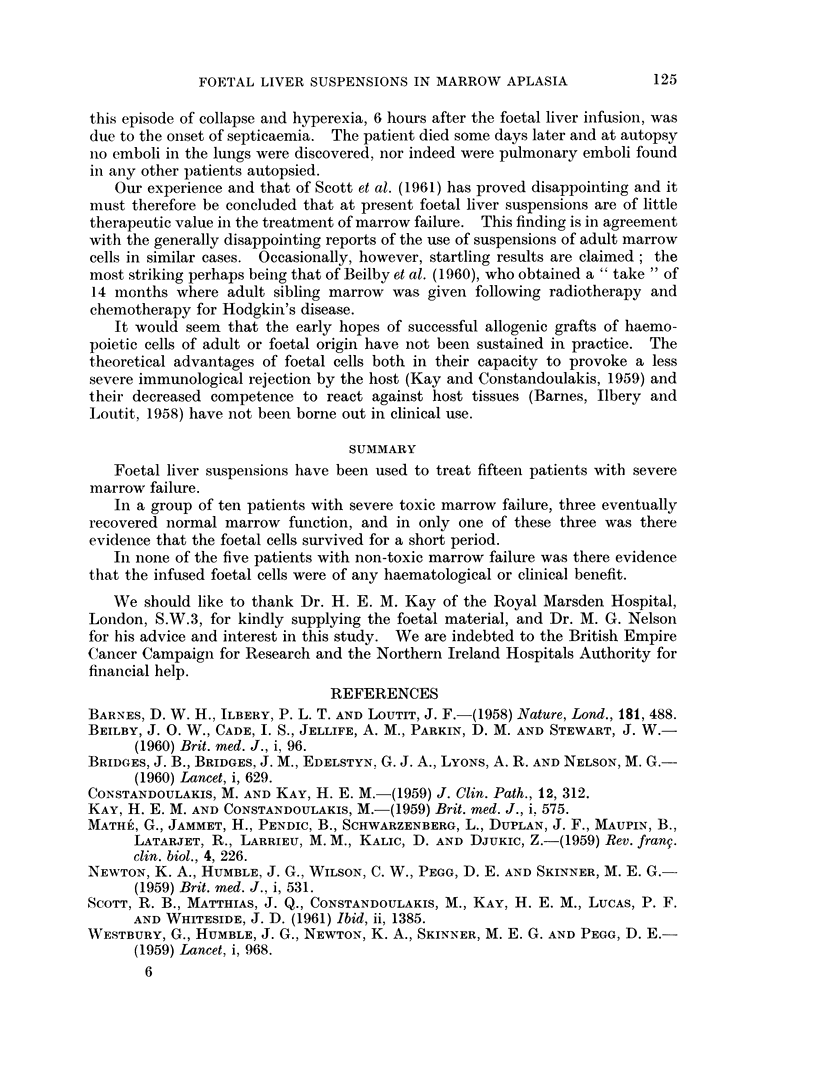# Foetal Liver Suspensions in the Treatment of Marrow Aplasia

**DOI:** 10.1038/bjc.1965.13

**Published:** 1965-03

**Authors:** J. B. Bridges, J. M. Bridges, G. J. A. Edelstyn, A. R. Lyons


### FOETAL LIVER SUSPENSIONS IN THE TREATMENT OF MARROW APLASIA

J. B. BRIDGES*, J. M. BRIDGES†, G. J. A. EDELSTYN‡ AND A. R. LYONS‡

*From the *Queens' University of Belfast, the †Royal Belfast Hospital for Sick Children, and the ‡Northern Ireland Radiotherapy Centre, Montgomery House, Belfast.*

Received for publication November 9, 1964

INFUSIONS of haemopoietic cells have now been widely used in the treatment of various forms of marrow failure. These cells have generally been obtained from an adult living donor (Mathé *et al.*, 1959), or from the patient before either chemotherapy (Westbury *et al.*, 1959) or radiotherapy (Newton *et al.*, 1959) for reinfusion after these procedures.

Kay and Constandoulakis (1959) have described a technique whereby the haemopoietic cells contained in the human foetal liver may be harvested, prepared, and stored in a form suitable for intravenous infusion. This communication describes the results obtained in treating fifteen patients suffering from marrow failure with this material.

### MATERIALS AND METHODS

The foetal liver suspensions were kindly supplied to us by Dr. H. E. M. Kay of The Royal Marsden Hospital, London. They were transported in sealed 5 ml. ampoules, stored in thermos flasks in a mixture of solid carbon dioxide and methylated spirits at $-79°$ C. Immediately before use the ampoule was quickly thawed and the material administered at a rate of 1 ml. per minute into an intravenous transfusion of blood, not more than 5 ml. amounts being given in any one hour. Each patient received between 18 and 25 ml. of this material except one who was given a total of 35 ml. in two divided doses with a 4-day interval.

Foetal liver infusions have been given in all to fifteen patients with severe marrow failure. The infusion of this material was possibly associated with an untoward reaction in only one patient suffering from toxic marrow failure. Six hours after the completion of the infusion she developed a severe pyrexial reaction accompanied by a rigor and circulatory collapse: the temperature rose from 99·6° F. to 105·6° F. and her blood pressure, previously 150/90 mm. Hg, fell to 80/40 mm. Hg. She was given 100 mg. hydrocortisone acetate by intravenous injection and within 8 hours the blood pressure and temperature readings had returned to their previous levels. There was no subsequent evidence of excess red cell breakdown to suggest that haemolysis of the transfused red blood cells had been the main cause of this reaction.

The patients receiving foetal liver suspension can be divided into two distinct groups. The first of ten patients developed marrow failure following exposure to cytotoxic drugs, and a second group of five patients in whom there was no known exposure to any toxic agent.

*Group 1.—Patients with toxic marrow failure*

These ten patients all had some form of neoplastic disease. Eight had disseminated carcinomatosis, five of mammary and three of bronchial origin; one had Hodgkin's disease; and one had lymphoid reticulosis. The three patients with bronchial carcinoma had been treated with T.E.M. (tri-ethylene melamine), while all the remaining patients had had intensive therapy with Thiotepa (tri-ethylene thiophoramide). Severe leuco- and thrombocytopenia (white cell count under 200 and/or platelet count below 40,000 per cubic mm.) was present in all these patients after completion of chemotherapy and persisted for at least 9 and usually 15–17 days preceding the foetal liver infusion. During the period of marrow aplasia neither steroid therapy nor transfusions with fresh blood produced any improvement.

In only three patients of this group of ten did the pancytopenia eventually resolve and the level of circulating cells reach pre-treatment levels. In the remaining seven cases there was no improvement in the pancytopenia before death 3–10 days following the foetal liver infusion. In each patient where genotype differences existed between the donor and the recipient's blood group differential agglutination studies were carried out using the modification of the Ashby technique described by Constandoulakis and Kay (1959). There was no firm evidence from these tests that red blood cells other than of recipient genotype were present in the peripheral blood of these patients. Post mortem examination was carried out in four of these patients and careful macroscopic and microscopic examination failed to show any evidence of pulmonary embolism. In each case there was extensive active malignant disease and the sequelae of marrow failure, including extensive petechial haemorrhages.

Of the three patients in whom the level of circulating cells reached pre-treatment levels, in no case was there any evidence that the infused cells survived permanently. In two patients there was no evidence of any clinical, or of direct haematological, benefit from the infused cells, but in the remaining patient there was evidence that the infused cells survived, matured and circulated for a short period. In this patient with mammary carcinoma the improvement in the clinical and haematological findings was immediate and striking following the foetal liver infusion, and normal levels of circulating cells were attained within 30 days. Examination of the patient's red and white cells in the post-infusion period suggested their origin in part from the infused cells and the detailed evidence for this conclusion has been published elsewhere (Bridges *et al.*, 1960). The white cell and platelet counts remained within normal limits for 24 months during which the carcinoma was quiescent and the secondary lesions improved and healed. The patient then relapsed and died 3 months later from generalised mammary carcinomatosis uncontrolled by a second course of Thiotepa.

In the other two patients with toxic marrow failure which resolved, no rise in the white cell or platelet counts occurred until 5 days following the infusion. There is no firm evidence that the infused foetal cells survived or contributed to the eventual marrow recovery. In these two patients both donor and host were of blood group O. In each transfer there were minor differences in genotype but blood transfusions given at the same time as the foetal liver made subsequent genotype analysis of red blood cells inconclusive.

*Group 2.—Patients with no known exposure to any toxic agent*

This group of five patients all had severe peripheral leuco- and thrombocytopenia at the time of foetal liver infusion. Two patients were in the terminal stages of Fanconi's familial aplastic anaemia ; one had idiopathic aplastic anaemia ; one was in the aplastic preleukaemic phase of monocytic leukaemia ; and one a terminal case of paroxysmal nocturnal haemoglobulinuria in the aplastic phase. None of these patients showed any untoward reaction to the foetal cell infusion. In no case was there any improvement in the clinical condition, or in the level of circulating blood cells, while differential agglutination studies showed no evidence of survival of infused cells of foetal origin.

In this group, four patients died within 7 days of the liver infusion. It was considered that the foetal liver suspensions did not contribute to these deaths as no evidence of pulmonary embolism was found at autopsy. The remaining patient survived for 3 months before death from acute monocytic leukaemia.

### DISCUSSION

The prognosis for patients with severe marrow failure is uncertain but, in general, if the marrow failure follows exposure to a toxic agent, then the outlook is somewhat better than if the marrow failure develops spontaneously. Therefore, in attempting to assess the value of infusions of foetal liver cells in our patients, we feel the two groups should be considered separately.

Of the group of ten patients with severe toxic marrow failure, three eventually recovered normal marrow function but in no case was there any evidence that the infused foetal cells survived permanently. In one patient there was evidence that the infused cells survived and matured, and that cells of foetal origin circulated in the patient's peripheral blood for a short period, and may have sustained her until normal marrow function was resumed. In the other two patients the possibility that some hormonal or nutritional factor was conveyed in the foetal infusions to these patients remains a possibility, without present proof of validity.

The outlook for patients with toxic marrow failure must depend not only on the degree of marrow failure but also on their general condition and presence of any other disease. In this regard it is important to note that our three patients who recovered marrow activity were the only ones whose malignant process had been favourably influenced by the chemotherapy given. In the other seven patients, whilst the immediate cause of death was haemorrhage or infection consequent on thrombocytopenia or leucopenia, the malignant process was disseminated and active and must have contributed substantially as a cause of death.

In none of our five patients with non-toxic marrow failure was the administration of foetal liver suspension of any value either clinically or haematologically. However, four of these patients were in the terminal stages of their illness at the time of infusion and the other died three months later following the development of frank monocytic blastic leukaemia.

Scott *et al.* (1961) have reported the results of giving foetal liver suspension to fourteen patients with idiopathic marrow failure and found it to be of temporary benefit in only two cases. These authors reported no untoward reaction in any of the patients. In the present series in only one instance was the administration of foetal liver suspension possibly associated with an adverse reaction. It may be this episode of collapse and hyperexia, 6 hours after the foetal liver infusion, was due to the onset of septicaemia. The patient died some days later and at autopsy no emboli in the lungs were discovered, nor indeed were pulmonary emboli found in any other patients autopsied.

Our experience and that of Scott *et al.* (1961) has proved disappointing and it must therefore be concluded that at present foetal liver suspensions are of little therapeutic value in the treatment of marrow failure. This finding is in agreement with the generally disappointing reports of the use of suspensions of adult marrow cells in similar cases. Occasionally, however, startling results are claimed; the most striking perhaps being that of Beilby *et al.* (1960), who obtained a " take " of 14 months where adult sibling marrow was given following radiotherapy and chemotherapy for Hodgkin's disease.

It would seem that the early hopes of successful allogenic grafts of haemopoietic cells of adult or foetal origin have not been sustained in practice. The theoretical advantages of foetal cells both in their capacity to provoke a less severe immunological rejection by the host (Kay and Constandoulakis, 1959) and their decreased competence to react against host tissues (Barnes, Ilbery and Loutit, 1958) have not been borne out in clinical use.

### SUMMARY

Foetal liver suspensions have been used to treat fifteen patients with severe marrow failure.

In a group of ten patients with severe toxic marrow failure, three eventually recovered normal marrow function, and in only one of these three was there evidence that the foetal cells survived for a short period.

In none of the five patients with non-toxic marrow failure was there evidence that the infused foetal cells were of any haematological or clinical benefit.

We should like to thank Dr. H. E. M. Kay of the Royal Marsden Hospital, London, S.W.3, for kindly supplying the foetal material, and Dr. M. G. Nelson for his advice and interest in this study. We are indebted to the British Empire Cancer Campaign for Research and the Northern Ireland Hospitals Authority for financial help.

### REFERENCES

BARNES, D. W. H., ILBERY, P. L. T. AND LOUTIT, J. F.—(1958) *Nature, Lond.*, **181**, 488.

BEILBY, J. O. W., CADE, I. S., JELLIFE, A. M., PARKIN, D. M. AND STEWART, J. W.—(1960) *Brit. med. J.*, i, 96.

BRIDGES, J. B., BRIDGES, J. M., EDELSTYN, G. J. A., LYONS, A. R. AND NELSON, M. G.—(1960) *Lancet*, i, 629.

CONSTANDOULAKIS, M. AND KAY, H. E. M.—(1959) *J. Clin. Path.*, **12**, 312.

KAY, H. E. M. AND CONSTANDOULAKIS, M.—(1959) *Brit. med. J.*, i, 575.

MATHÉ, G., JAMMET, H., PENDIC, B., SCHWARZENBERG, L., DUPLAN, J. F., MAUPIN, B., LATARJET, R., LARRIEU, M. M., KALIC, D. AND DJUKIC, Z.—(1959) *Rev. franç. clin. biol.*, **4**, 226.

NEWTON, K. A., HUMBLE, J. G., WILSON, C. W., PEGG, D. E. AND SKINNER, M. E. G.—(1959) *Brit. med. J.*, i, 531.

SCOTT, R. B., MATTHIAS, J. Q., CONSTANDOULAKIS, M., KAY, H. E. M., LUCAS, P. F. AND WHITESIDE, J. D. (1961) *Ibid*, ii, 1385.

WESTBURY, G., HUMBLE, J. G., NEWTON, K. A., SKINNER, M. E. G. AND PEGG, D. E.—(1959) *Lancet*, i, 968.